# The Hamburg integrated medical degree program iMED

**DOI:** 10.3205/zma001260

**Published:** 2019-10-15

**Authors:** Anke Rheingans, Athanasios Soulos, Sonja Mohr, Jelka Meyer, Andreas H. Guse

**Affiliations:** 1 University Medical Center Hamburg-Eppendorf, Faculty of Medicine, Dean's Office for Student Affairs, Hamburg, Germany; 2 University Medical Center Hamburg-Eppendorf, Department of Biochemistry and Molecular Cell Biology, Hamburg, Germany

**Keywords:** medical degree, integrated curriculum, scientific orientation, practical skills, communicative competencies, interprofessional education

## Abstract

The integrated medical degree program (iMED) was established in winter semester 2012/2013 at the Faculty of Medicine of Universität Hamburg with the aim of improving medical education. The main features of the iMED medical degree program include the close integration of theoretical knowledge and practical skills, scientific orientation and the teaching of psychosocial and communication skills. All these features are commonly found in the modular compulsory core curriculum and elective courses (“2nd Tracks”): The compulsory core curriculum comprises 19 modules which are arranged thematically in seven module groups and cover three stages of a “learning spiral”. By comprehensively coordinating the teaching content and the learning objectives of the participating theoretical and clinical subjects, theoretical content is taught on the basis of real patient’s medical histories from the first stage of the learning spiral. The elective courses enable students to learn and apply scientific work in a structured curriculum according to their own interests. Relevant practical skills for students future professional routines are taught in the longitudinal training course “Clinical Examination Methods plus Communication” (KUMplusKOM), which runs through the entire curriculum up to the final practical year. Accompanying, extra-curricular projects such as crash courses in the natural sciences or using the iMED Textbook as an online learning platform increase the attractiveness of the iMED degree program. Results of the evaluation show that the introduction and the accompanying optimization of iMED were very successful.

## Zusammenfassung

Der integrierte Modellstudiengang Medizin (iMED) wurde zum Wintersemester 2012/2013 an der Medizinischen Fakultät der Universität Hamburg mit dem Ziel der Verbesserung der ärztlichen Ausbildung initiiert. Zu den wesentlichen Merkmalen des Modellstudiengangs iMED gehören eine enge Verzahnung von theoretischem Wissen und praktischen Fertigkeiten, wissenschaftliche Orientierung sowie die Vermittlung psychosozialer und kommunikativer Kompetenzen. Alle Merkmale finden sich vielfach in dem modular aufgebauten Pflicht- und Wahlpflichtcurriculum wieder: Das Pflichtcurriculum besteht aus 19 Modulen, die in sieben Modulgruppen thematisch angeordnet sind und sich über drei Stufen einer Lernspirale erstrecken. Durch eine umfangreiche Abstimmung der Lehrinhalte und Lernziele der beteiligten theoretischen und klinischen Fächer werden theoretische Ausbildungsinhalte bereits ab der ersten Stufe der Lernspirale anhand von Beispielerkrankungen und realen Krankheitsgeschichten vermittelt. Der Wahlpflichtbereich ermöglicht den Studierenden nach eigener Interessenslage wissenschaftliches Arbeiten in einem strukturierten Mantelcurriculum zu erlernen und anzuwenden. Für den beruflichen Alltag relevante praktische Fertigkeiten vermittelt der longitudinale Ausbildungsstrang klinische Untersuchungsmethoden plus Kommunikation, der sich durch das ganze Curriculum bis zum Praktischen Jahr zieht. Flankierende, außercurriculare Projekte wie z. B. Crash-Kurse in den Naturwissenschaften oder iMED Textbook als online-gestützte Lernplattform erhöhen die Attraktivität des iMED-Studiengangs. Wie die Ergebnisse der studentischen Lehrevaluation zeigen, sind der Einstieg und die begleitende Optimierung des Modellstudiengangs iMED aus Sicht der Hamburger Medizinischen Fakultät sehr erfolgreich verlaufen.

## 1. Introduction

The integrated medical degree program in medicine (iMED) was created and prepared between 2008 and 2012. Lecturers from all disciplines of the Faculty of Medicine of Universität Hamburg and the University Medical Center Hamburg-Eppendorf have developed the degree program together with students in a large-scale work and coordination process. The results of the 2007/08 graduate survey provided the initial key impetus for the process. In particular, responses to the survey indicating that respondents felt that practical skills were inadequately taught and that a lack of scientific training was evident [1]. The Bologna Process also influenced the development of iMED and led to the design of a modular degree program. The first cohort of students was admitted in winter semester 2012/2013. The degree program was approved by the responsible authority in accordance with federal state law until 2022.

## 2. iMED integrated medical degree program – concept and goals

### 2.1 Reform goals, central guiding principle and structural goals

The overriding goals of iMED are the significant improvement of medical education and the visualization of teaching innovations in medicine. iMED is characterized by the guiding principle of “scientific orientation”. The Faculty of Medicine expects its graduates to adopt a questioning and critical attitude, to be able to structure themselves and be independent, as well as pursuing a consistent orientation towards evidence-based science. Central tasks of equal value include the teaching of social skills as well as practical medical skills. Another important goal of the study reform is the long-term engagement of excellent young physicians.

The structural goals are the integration of theoretical and clinical training content, the subdivision into a core curriculum which is supplemented with an extended elective study program as well as a higher degree of self-determined teaching and learning. Counselling services and measures aimed at qualifying lecturers form an important basis for the degree program, alongside other quality assurance measures [2].

#### 2.2 Curriculum structure

**Figure 1:**
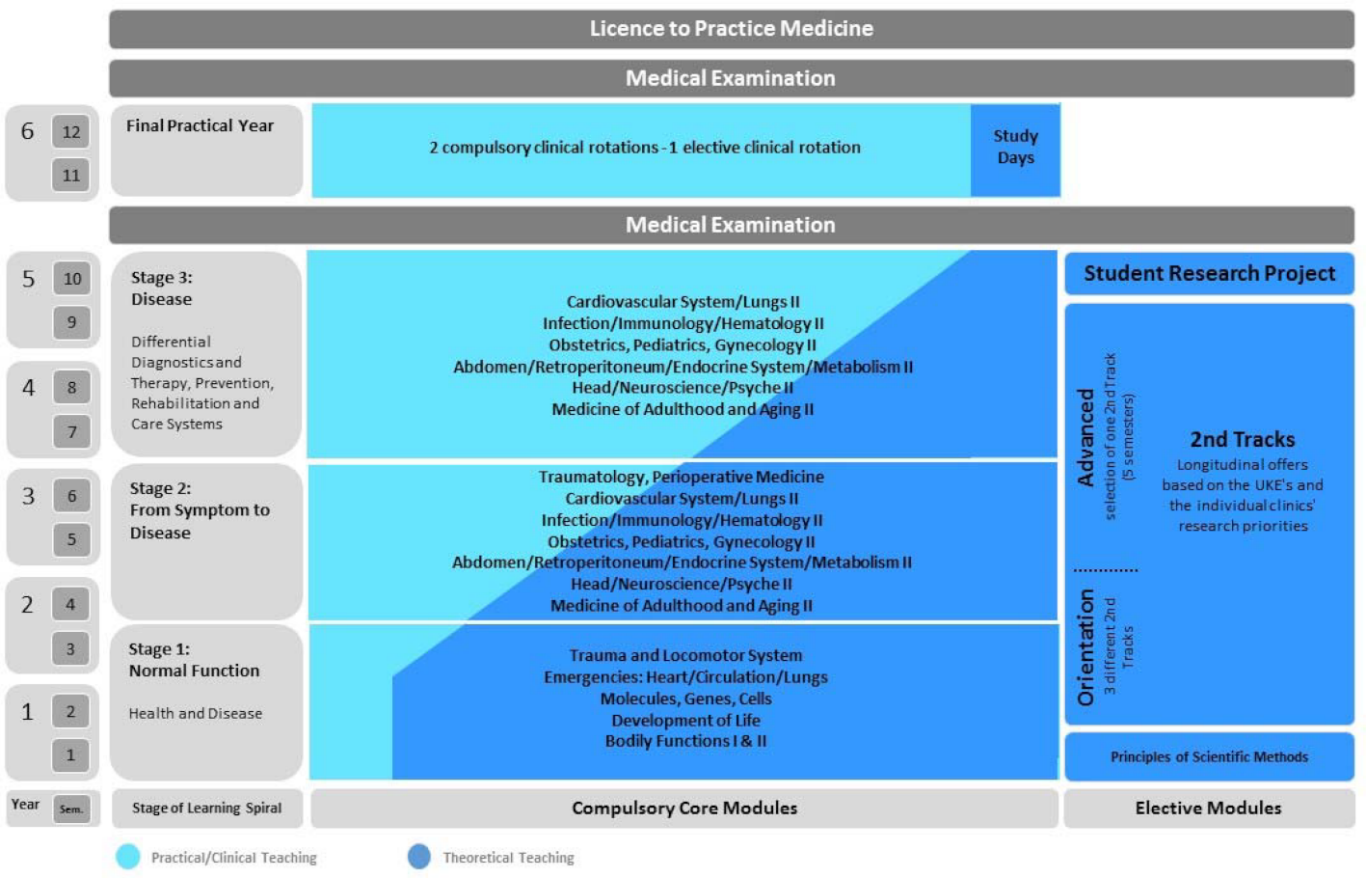
Schematic illustration of the iMED degree program (based on [2])

iMED consists of a modular compulsory core curriculum and elective courses. The core curriculum is comprised of 19 modules which are arranged in seven module groups and cover three stages of a learning spiral. The elective modules are thematically structured, longitudinally arranged and underline the scientific aspects of medicine. The first nine semesters consist of two six-week compulsory modules and one two-week elective module (2^nd^ Track).The intermediate examination (designed as a university examination) “Normal function: health and disease” is taken at the earliest after the third semester. In the tenth semester, students prepare a student research project as an independent academic achievement. After the tenth semester, the “Second Part of the Medical Examination” is taken. This is followed by the final practical year. The medical degree program is completed with the “Third Part of the Medical Examination” (cf. figure 1 (Fig. 1)) after six years and three months at the earliest. 

The compulsory core curriculum is divided into three large sections in the form of a three-stage learning spiral with increasing requirements: “Normal function: Health and disease” (semesters 1-3), “From symptom to disease” (semesters 4-6) and “Disease – differential diagnosis and differential therapy, prevention, rehabilitation and care systems” (semesters 7-9). In each of these three stages, closely related or similar topics, which are nevertheless progressive in their complexity, are gradually dealt with in greater depth in seven compulsory module blocks.

The Z-shaped structure of iMED illustrated in figure 1 (Fig. 1) underlines the close linkage between theoretical (dark blue) and practical-clinical teaching components (light blue). By comprehensively coordinating the teaching content and the learning objectives of the participating theoretical and clinical subjects, theoretical training content is taught on the basis of real patient’s medical histories from the first stage of the learning spiral. In this way, students do not only learn the theoretical contents in the systematics of each individual subject, but also in a comprehensive way that references the respective leading diseases. One teaching innovation that students rated very positively in this context is team teaching. This teaching format includes collaborative teaching by lecturers from basic medical sciences and from clinical subjects regarding a disease, including the involvement of patients in several lectures. 

#### 2.3 Scientific principles as a guiding principle

The compulsory elective course (2^nd^ Track) enables students to learn and apply scientific work in a structured curriculum. Accordingly, a wide range of 2^nd^ Tracks was established oriented towards the Faculty's main research areas, the UKE's clinical interests and the interests of the students. The consecutive 2^nd^ Tracks from the 1st to the 9th semester aim at the structural integration of scientific approaches in concordance with theoretical and clinical topics in various areas of medicine. Currently, 18 tracks, for example, dealing with the “Importance of Genetics in Prenatal Diagnosis and Pediatrics”, "Inflammation, Infection, Immunity" and "intermed - Intercultural Competence and International Medicine" (a current list can be found on https://www.uke.de/studium-lehre/modellstudiengang-medizin-imed/).

In the first semester of the elective course, all students must first complete the module “Principles of Scientific Methods”. These knowledge will be addressed and expanded over the course of the 2^nd^ Tracks to ensure the longitudinal integration of the guiding principle. The 2^nd^ Tracks are completed in the tenth semester by conducting an independent student research project, which is compulsory for all students. The student research project is a descriptive, theoretical and literature-based project; practical or empirical parts are only accepted in exceptional cases. The project is graded by two teachers (associate or full professors) using a standardized evaluation sheet with predefined criteria. It is possible to thematically combine a student research project and a medical dissertation, however, two independent papers must be written and submitted. This motivates students to start working on an eligible medical dissertation.

#### 2.4 KUMplusKOM framework (Clinical Examination Medicine plus Communication)

At the end of their studies, iMED graduates should be able to establish a complete, structured and individual anamnesis of the patient empathetically and efficiently as well as carrying out a comprehensive physical examination and prescribing suitable diagnostic measures. Furthermore, they should also be able to independently carry out simple interventional measures, conduct patient-centered consultations, initiate therapeutic measures and develop therapy concepts in collaboration with patients and relatives. 

**Figure 2:**
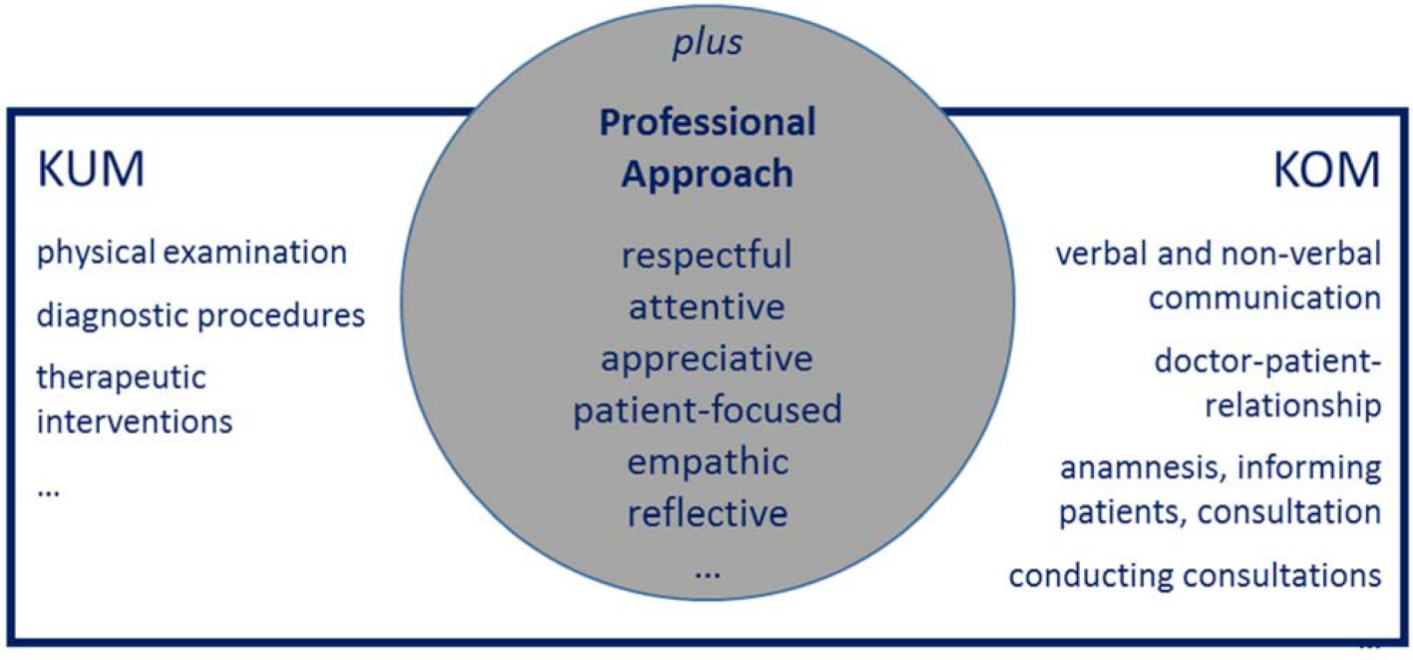
KUMplusKOM framework

The longitudinal training course "KUMplusKOM" is integrated in several modules and contributes both to the acquisition of practical competencies in the field of clinical examination methods (KUM) and to the development of communicative competencies in discussions with patients, relatives and colleagues (KOM) (see figure 2 (Fig. 2)).

## 3. Assessment strategy, structure and organization

The iMED assessment strategy comprises 19 compulsory module examinations, nine compulsory elective module examinations plus the compulsory elective module "student research project" as well as an oral/oral-practical intermediate examination following the third semester (“Examination Normal function: Health and disease”). A module examination consists of a final module examination for the respective module and optional course performances during the semester. As with the teaching content, the module examinations were designed to be cross-curricular by the teachers of the above-mentioned module groups on the basis of the intended learning outcomes. In seven compulsory modules objective structured clinical examinations (OSCE) were introduced to test the clinical-practical decision-making and responsibility. The transition from semester examinations to module examinations is differently received: While the students consider the 6-2-6 week rhythm (compulsory module-2ndTrack-compulsory module) to be useful for exam preparation, the teachers are rather critical of the increased deadline pressure and the accumulation of exams.

Of the three state examinations stipulated in the current Licensing Regulations for Physicians in Germany (Ärztliche Approbationsordnung, ÄApprO, 27^th^ June 2002), exempt the “First Part of the Medical Examination”: The successful completion of the module examinations for the first eleven compulsory modules after the fifth semester forms the equivalence to the written part of the “First Part of the Medical Examination” (§ 41 Paragraph 1 Number 1 ÄApprO). The “Examination – Normal function: Health and Disease” replaces the oral-practical part of the “First Part of the Medical Examination” as an oral/oral-practical intermediate examination after the third semester. The oral part of the "Examination – Normal Function" covers the preclinical sciences anatomy, biochemistry, physiology and medical psychology/medical sociology. One week before their examination date, students are notified in which two of the four subjects they will be tested. The oral-practical part takes place as an OSCE with twelve stations. The implementation of these exams requires a high workload of the teaching staff (examiners and supervisors) and of the Dean's Office for Student Affairs. Passing the “Examination – Normal Function” and the first eleven compulsory modules is a prerequisite for continuing the iMED program beyond the fifth semester. 

## 4. Accompanying measures and offers

Success in higher education is not only determined by subject-specific factors such as innovative curricula. The selection of suitable applicants and a supportive learning environment also facilitate successful education and increase the attractiveness of the iMED medical degree program. 

### 4.1 Before the study – student selection at Hamburg Medical Faculty

At present, a multi-stage selection procedure is being carried out which focuses on previous scientific knowledge in a multiple-choice test (HAM-NAT, 80 questions) as well as on psychosocial and communicative competences in a multiple mini-interview (HAM-INT) [3], [4]. The HAM-NAT natural science test in particular has already demonstrated an efficient predictive ability to determine academic success in preclinical sciences [5]. The research group is currently developing proposals for new selection procedures and simulating their effects on the basis of data from applicants in Hamburg [6].

#### 4.2 During the study 

##### 4.2.1 “iMED Crash Courses” in natural sciences 

In order to address the fact that (too) many students in their first semesters have little or no scientific background knowledge, extra-curricular “crash courses in natural sciences” are offered in chemistry, biology, physics and mathematics. These courses are integrated in the first semesters so that they can optimally prepare the students for the curricular courses and thereby improving their academic performance [7]. A total of 32 crash courses are offered as part of the iMED program: 13 chemistry crash courses in the first, second and third semesters, twelve physics crash courses in the first, third and sixth or seventh semesters respectively, four biology crash courses in the second semester and three mathematics crash courses in the first semester. All of the crash courses in the natural sciences that have been taught so far have been rated good to very good by the students.

##### 4.2.2 iMED-Textbook online learning platform

The integrated curriculum challenges the students to master the simultaneous development of the subject matter required for the preclinical sciences as well as the clinical subjects. In order to provide students with an easy and uncomplicated introduction, the online learning and working platform "iMED Textbook" was developed as a tailor-made, multimedia and interactive eLearning concept. The iMED Textbook is linked to the learning objectives database and comprises over 25,000 standard text pages (including over 6,000 illustrations and tables) [8], [9].

##### 4.2.3 Interprofessional education 

An annual elective course was established in winter semester 2016/17 as an interprofessional pilot project with a clinical focus in the compulsory elective course curriculum. Medical students work together with nursing students to investigate real patient cases and identify common ground between the two professions as well as the patient benefits generated by an interprofessional approach. The accompanying evaluation indicated high scores in the overall satisfaction (6-point Likert scale; 1 “strongly disagree” to 6 “strongly agree”) of the participants (n=88, M=4.97, SD=1) in all semesters (winter semester 2016/17, 2017/18, 2018/19).

#### 4.3 Beyond the degree program

##### 4.3.1 Mentoring program

The “iMED Mentoring” program is a differentiated advisory and support program which is open to all students (general mentoring program). The annual increase in enrolment rates from approx. 50% to over 60% since the start of the program demonstrates the steadily increasing popularity of the program among students. Furthermore, two special programs support both high-performing and low-performing students [10]. To ensure the success of these programs, the matching procedures of the mentor and mentee, for which individual procedures have been developed, are particularly important [11], [12].

## 5. Six years in iMED: experiences and success

### 5.1 Successful quality assurance

**Figure 3:**
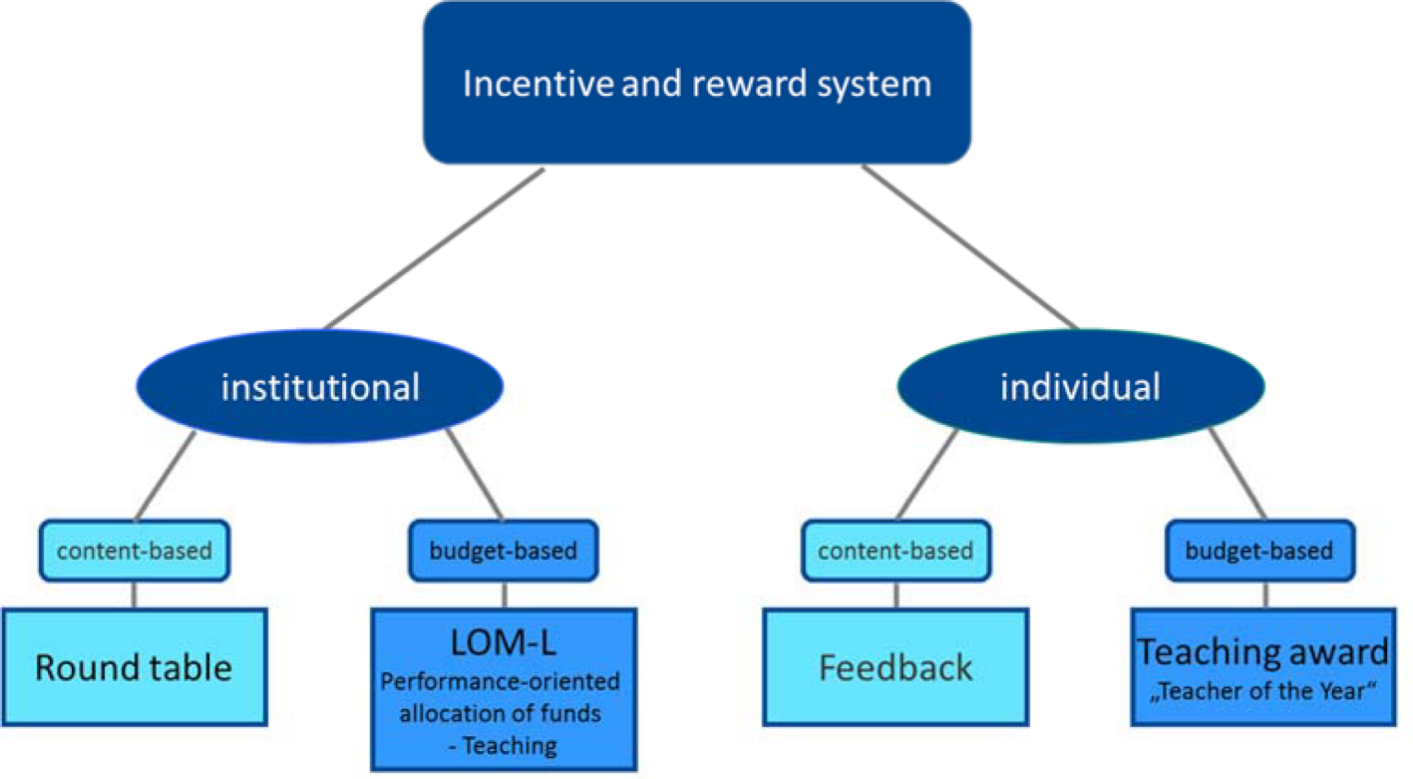
Incentive and reward system "for good teaching" at the Faculty of Medicine, Universität Hamburg

For many years, the Faculty of Medicine has applied a system of incentives and rewards for teaching (see figure 3 (Fig. 3)), which is based on the evaluation of teaching. This was adapted and expanded to incorporate the design of iMED. The basis is the evaluation of the courses attended by the students, however, their satisfaction with the overall design of the module as well as proposals for awarding a teaching prize are also included. The results are made available to various committees at an institutional level, e.g. the iMED Curriculum Committee (CK iMED), as part of a round table and used for the ongoing optimization of the curriculum. This committee played an important role in the content design and fine-tuning, particularly during the degree programs initial stages. For example, first-year students criticized the late and unclear presentation of intended learning outcomes in modules of the first three semesters. “Learning objectives were helpful for structuring the subject matter”: 2.31 [21.8% satisfaction]). The subsequent intensive analysis of the learning objectives by the teachers involved led to an increase in the rating to 4.21 (75.8% satisfaction) in the second year of the degree program (summer semester 2014). Financial incentives are provided via the performance-oriented allocation of funds based on teaching quality. In particularly difficult situations encountered during teaching, individual discussions are held to improve the teaching process. Every year, students commend particularly dedicated teachers with the “Teacher of the Year” award. 

**Figure 4:**
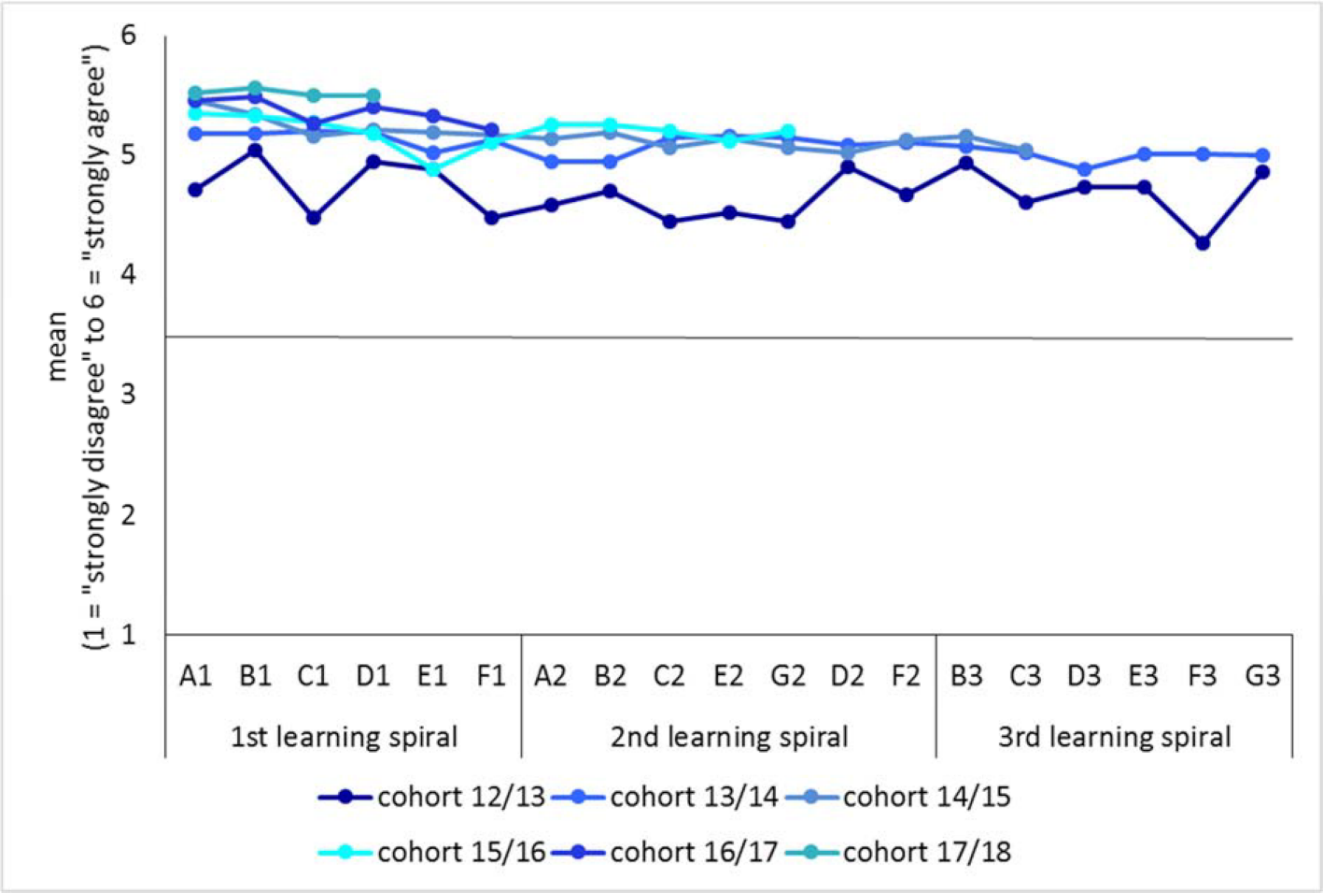
Satisfaction with the entire iMED degree program according to academic year (summer semester 2018)

The aggregated evaluation data for the modules carried out to date in the first, second and third learning spiral (see figure 4 (Fig. 4)) demonstrate very clearly that the targeted measures to improve quality have already led to a significant improvement in overall satisfaction from the second year onwards (cohort 2013/14). A Mann-Whitney-U-test shows that the student satisfaction in the degree program (n=18913 evaluations: M=5.11, SD=0.91, Mdn=5.00) is significantly higher than in the standard degree program (n=7510 evaluations): M=4.18, SD=1.15, Mdn=4.00; U=36850300.5, z=-64.59, p<.001, r=-.40). The number of evaluations is higher for iMED because the number of surveys during the degree program is significantly higher than for the standard degree program.

The high level of student satisfaction with iMED is also reflected in many free text comments. Students belonging to all academic years clearly identify the (few) content-related and organizational problems, enabling CK iMED and the module working groups to precisely address the problems that still exist or are about to arise. 

The evaluation results after six years of iMED also demonstrate that the core objectives of the model degree program, such as the integration of theoretical and clinical contents, the early integration of clinical subjects combined with early patient contact, scientific orientation, but also emphasizing medical-practical skills and psychosocial competences, are very well received.

#### 5.2 Needs and requirements of the integrated curriculum

In retrospect, the integration of theoretical and clinical training content (the central principle in the iMED curriculum), has posed a variety of challenges for both the teaching and administrative staff. First of all, a change of perspective had to be implemented, in which the subject-centered perspective was withdrawn and, subsequently, an interdisciplinary, content-oriented perspective was adopted. In order to make this change possible, structures to facilitate interdisciplinary exchange were established within the faculty. These included, among other things, retreats for the entire faculty, interdisciplinary module meetings and the iMED curriculum committee. The subjects had to adjust to distributing their teaching content over several semesters and, if necessary, taking on a dominant role in one module and a supporting function for other subjects in another module. The challenge here was to master the coordination within the discipline and with other subjects. Accommodating the students according to their level of knowledge and neither over- nor under-challenging them in class or in examinations remains an important element of the coordination process.

The reduction in the autonomy of subjects posed unique challenges on the administration. A work interface was developed incorporating a learning objectives database and the iMED-Campus administration tool.

Without appropriate IT-supported assistance, the administration of the complex curriculum – especially timetabling and room management – would neither be feasible nor comprehensible. This professionalization of the administration has also resulted in a quantitative and qualitative change of staff. Overall, the reform process has led to further reforms both at subject level and in the program administration. A positive effect of overcoming these challenges is the greater understanding of each other's needs.

## 6. Conclusion

All in all, the introduction and the accompanying optimization of the iMED degree program went very well: First-year students (winter semester 2012/2013) successfully participated in the “Second Part of the Medical Examination” for the first time in autumn 2017 and spring 2018 – with a success rate of 100 percent – and went on to continue their studies in the “Final Practical Year”. The overall results for the first iMED cohort in the “Second State Examination” are comparable to those of the former cohorts in the standard medical degree program. In the nationwide ranking of the medical faculties, the Faculty of Medicine in Hamburg remains at a constant level with this result. The differences between faculties in the ranking are very small and only account for a few percentage points, though. Also, better results in the “Second State Examination” were not among the objectives when designing the degree program. The central focal points and strengths of the integrated curriculum are the integration of theory and practice, the early implementation of practical teaching and the emphasis on scientific education. These aspects are only scarcely reflected in the “Second Part of the Medical Examination”. The first iMED graduates took up employment in spring 2019.

## Competing interests

The authors declare that they have no competing interests.
